# In vitro digestion models in food chemistry: advancements, challenges, and applications in nutrient bioaccessibility and bioavailability

**DOI:** 10.1016/j.fochx.2026.103951

**Published:** 2026-05-06

**Authors:** Farhang Hameed Awlqadr, Mohammed N. Saeed, Othman Abdulrahman Mohammed, Syamand Ahmed Qadir, Aryan Mahmood Faraj, Seyed Mohammad Najibi Hosseini, Muhammad Tayyab Arshad, Muhammed Adem Abdullahi, Tablo H. Salih

**Affiliations:** a Food Science and Quality Control, Halabja Technical College, Sulaimani Polytechnic University, Sulaymaniyah, Iraq; b Department of Nutritional Analysis and Health, Kifri Technical College, Garmian Polytechnic University, Kifri City, Sulaimaniyah Province, Kurdistan of Iraq, Iraq; c Medical Laboratory Science Department, Halabja Technical College, Sulaimani Polytechnic University Sulaymaniyah, Iraq; d Medical Laboratory Techniques Department, Halabja Technical Institute, Research Center, Sulaimani Polytechnic University, Sulaymaniyah, Iraq; e Department of Animal Biology, Faculty of Natural Sciences, University of Tabriz, Iran; f Department of Food Science and Technology, Faculty of Agriculture, University of Tabriz, Iran; g Functional Food and Nutrition Program, Faculty of Agro-Industry, Prince of Songkla University, Hatyai, Songkhla 90110, Thailand; h Department of Food Science and Postharvest Technology, Jimma University College of Agriculture and Veterinary Medicine, Jimma University, Jimma, Ethiopia

**Keywords:** In vitro digestion models, Nutrient bioaccessibility, Nutrient bioavailability, Static and dynamic models, Caco-2 cells, Organ-on-chip, OMICS technologies

## Abstract

Modern food chemistry and nutrition research stresses digestion and bioavailability. In vitro digestive models are needed to reconstruct the human gastrointestinal (GI) system and study food-derived chemical release, transformation, and absorption. This review examines major in vitro digestion platforms, including static and dynamic systems, organ-on-chip devices, and 3D-bioprinted gut models. Integrating these models with absorption simulators such as Caco-2 cells and advanced analytical instruments (e.g., HPLC, LC-MS/MS, FTIR, NMR, and OMICS technologies) enables comprehensive profiling of digestion products. These models assess bioaccessibility and bioavailability of polyphenols, omega-3 fatty acids, and encapsulated nutraceuticals. Computer modeling and artificial intelligence (AI) increase prediction for high-throughput functional food performance screening. Though promising, current models lack homogeneity and microbial representation often does not fully replicate in vivo gut microbiota complexity. In vitro digestive models assist food scientists enhance products. Priorities include harmonizing INFOGEST protocols, improving physiological relevance, and customizing nutrition and clinical validation. These developments may strengthen the utility of in vitro models in precision nutrition and industrial applications.

## Highlights

• Review of key static and dynamic in vitro digestion models.
• Comparison of model accuracy in mimicking GI conditions.
• OMICs tools boost understanding of digestion mechanisms.
• Analytical methods track nutrient release and changes.
• Advances in gut-on-chip and 3D models for relevance.

## 1 Introduction

Food digestion is a series of chemical reactions that involve the physical breaking of food, the enzymatic breakdown and the change of the molecules to smaller ones in the digestive tract. Knowledge about the release of nutrients and bioactive compounds from food under physiological conditions is a prerequisite for the evaluation of their nutritional and health-promoting properties. This knowledge has become the center of attention in modern food chemistry, which connects the gap between food composition and the way it is metabolized by the human body (Capuano & Janssen, 2021). Typically, the understanding of digestion and nutrient absorption has been based on the results of animal and human in vivo studies. Nevertheless, the development of in vitro digestion models has substantially advanced research on food functionality, nutrient release, and bioavailability, thus providing more ethical, cost-effective, and standardized alternatives (Bohn et al., 2018). However, they also entail several drawbacks. The controversies over the use of humans and animals for experiments, variability among subjects, elevated expenses, protracted periods of research, and challenges in managing the parameters of the control group are some of the constraints impeding their practicability (Abuhassira-Cohen & Livney, 2022; Mota et al., 2023). Moreover, in vivo techniques are often limited in their ability to provide mechanistic insights on the biochemical changes of food components in the gastrointestinal (GI) tract. In vitro digestion models simulate sequential digestive events and are widely used to investigate nutrient release, transformation, and predicted absorption. They enable nutrient breakdown monitoring, identifying digestion intermediates, conducting studies on bioaccessibility (the portion of a compound released from the food matrix and available for absorption), and predicting bioavailability when used along with cellular absorption models (Ji et al., 2022; Minekus et al., 2014). By design, researchers achieve precise control of pH, enzyme concentrations, digestion duration, and mechanical mixing, making these models highly suitable for reproducible and mechanistic studies in food chemistry and nutrition. The invention of the INFOGEST static in vitro digestion protocol, developed as an international consensus method by the INFOGEST network (COST Action FA1005), has been a major milestone and is now the most widely used harmonized protocol for simulating gastrointestinal digestion in vitro (Brodkorb et al., 2019). Despite the wide adoption of the INFOGEST protocol, several practical challenges remain that limit its cross-laboratory reproducibility. One major source of variability arises from differences in enzyme sources and activities, especially for pepsin, pancreatin, and bile extracts, which may vary between suppliers or batches, leading to inconsistent proteolysis or lipolysis rates across laboratories (Bohn et al., 2018; Brodkorb et al., 2019). In addition, pH control during gastric and intestinal phases often introduces discrepancies, as slight variations in titration procedures, electrode calibration, or buffering capacity of the food matrix can alter enzyme activity and digestion kinetics (Verkempinck et al., 2022). Another critical factor is operator-dependent variations, such as mixing intensity, timing, temperature equilibration, and sample handling, which can significantly influence micelle formation, protein hydrolysis patterns, and bioaccessibility outcomes (Tan et al., 2022). Recent studies have therefore emphasized the need for harmonized reporting, enzyme activity normalization, and inter-laboratory ring trials to improve reproducibility and ensure that INFOGEST-based results can be reliably compared across institutions (Freitas et al., 2025). Addressing these practical issues is critical for strengthening the global utility of the INFOGEST protocol in food chemistry and nutrition research. In addition to enabling direct comparison across studies, such standardization also facilitates collaborative research between universities and industry. Moreover, the consortium of the INFOGEST has also established protocols for infants, the elderly, and people with impaired digestion, which shows the flexibility of the model for different physiological conditions further (Egger et al., 2016; Zhou et al., 2023). In vitro digestion is a major research area that could be effectively combined with analytical and omics technologies. The employment of metabolomics, proteomics, lipidomics, and transcriptomics alongside digestion models enables a system-level understanding of the changes occurring during digestion. For instance, mass spectrometry and nuclear magnetic resonance (NMR) are methods that can quickly pinpoint digestion intermediates and metabolites (Madalena et al., 2022; Smeets et al., 2021). These data indicate the changes in nutrient release and transformation that are the result of the composition, structure, and processing of food, which is an area of research that is rapidly expanding due to the development of functional foods and personalized nutrition programs.

Moreover, combining in vitro digestion with cellular absorption models such as Caco-2 monolayers or intestinal organoids provides an initial indication of the intestinal transport of nutrients and bioactive compounds. This integration helps bridge bioaccessibility and bioavailability and provides a more detailed understanding of nutrient function (Antal et al., 2024; Rodrigues et al., 2022). These hybrid structures are still under development; however, they show potential as alternative platforms to reduce reliance on animal models. In addition, they may provide more humane, cost-effective, and scalable approaches for evaluating drug and food absorption. In vitro digestion models play an important role in nutrient research and are increasingly applied in food product development, fortification strategies, bioavailability enhancement, nutraceutical design, and food contaminant risk assessment (Lesmes, 2023; Marze, 2017). Researchers, for instance, have used a simulated digestion technique to measure the influence of encapsulation on curcumin release, the impact of processing on protein digestibility, and the effect of lipid formulation on omega-3 bioaccessibility. The use of digestive models is growing to anticipate the interactions between food and gut microbiota, especially through the visualization of colonic fermentation (Shah et al., 2016; Young et al., 2020). One problem with the current models is that they frequently oversimplify the gastrointestinal complexity and lack some characteristics, for example, mucus barriers, immune interactions, peristaltic motion, or the fluctuating presence of gut microbiota. Besides, while standardized models such as INFOGEST serve as good benchmarks, the differences in enzyme activity, pH profiles, and digestion duration from one laboratory to another can still lead to variations in results. Further development of dynamic, physiologically relevant digestion systems such as TIM-1, SHIME, and Tiny-TIM remains necessary, as these platforms provide real-time control of pH, flow rates, and secretions to better simulate human digestion (Dupont et al., 2019; Singh, 2024). On the other hand, these sophisticated models have raised the complexity and the costs, which can make them less accessible for the usual use. Against this background, this review summarizes the current state of in vitro digestion models in food chemistry. We also discuss future directions in in vitro digestion research, including integration with OMICS, dynamic simulation, and cell-based absorption models. By outlining key improvements, applications, and limitations, this review provides a framework for understanding how in vitro digestion models may further advance food science and human nutrition.

## 2 Overview of in vitro digestion models

The development of in vitro digestion research began with simple enzymatic assays in the 1970s, which typically used isolated enzymes such as pepsin or pancreatin to estimate protein or lipid digestibility (Chernukha et al., 2021). These early approaches were useful but lacked physiological realism because they did not simulate sequential phases, pH transitions, or gastrointestinal transit. As the need for more standardized and comparable methods grew, researchers introduced multi-step static models, which laid the groundwork for the creation of the internationally harmonized INFOGEST static digestion protocol, now widely adopted due to its reproducibility and consensus-based design (Brodkorb et al., 2019; Minekus et al., 2014). In parallel, engineering advances supported the development of dynamic gastrointestinal simulators such as TIM-1 and SHIME, which incorporate peristalsis, secretion kinetics, and time-dependent pH changes to better mimic human physiology (Blanquet et al., 2004; Zhu et al., 2024). Together, these developments illustrate how the field evolved from basic biochemical assays to sophisticated models capable of capturing realistic digestive processes.

### 2.1 Static vs. dynamic models

The comparison between static and dynamic in vitro digestion models is a central topic in food chemistry research, especially when the models are applied to evaluate the bioaccessibility and bioavailability of nutrients. Static models are relatively straightforward and economical systems that mimic the digestive processes by sequentially exposing food samples to standard or controlled digestive fluids (saliva, gastric juice, and intestinal fluids) at pre-set pH, temperature, and enzyme concentrations. Such models as well as the globally standardized INFOGEST protocol developed by the COST Action Network, provide a harmonized approach to measuring the digestibility of proteins, carbohydrates, and fats as well as the release of bioactive compounds (Colombo et al., 2021; Faubel et al., 2023). The consensus method of INFOGEST facilitates not only the reproducibility but also the possibility of comparison between different laboratories, as it sets out physiologically relevant conditions including enzyme activities (e.g., pepsin and pancreatin), bile salts, electrolyte composition, and timing for each phase (Colombo et al., 2021). Static models are predominant as they are more accessible and scalable and thus suitable for the preliminary studies of functional ingredients, encapsulation systems, and fortified foods (Minekus et al., 2014; Tan et al., 2022). However, static models still have a deficiency of some physiological complexities, such as peristalsis, gradual pH changes, continuous secretion of digestive fluids, and absorption, which are essential characteristics for the closely mimicking of the in vivo gastrointestinal (GI) tract (Alegría et al., 2015).

Dynamic models were developed to address these limitations and to more accurately reproduce gastrointestinal functions. Two representative dynamic systems are TNO's TIM-1 (TNO Intestinal Model-1) and SHIME (Simulator of the Human Intestinal Microbial Ecosystem), which simulate mechanical movements, secretion kinetics, and gradual pH changes over time to provide greater physiological relevance (Minekus, 2015; Zhu et al., 2024). For example, TIM-1 can be used for the description of the dynamic side of gastric emptying and small intestinal transit and has been confirmed to make a prediction of the bioaccessibility of lipophilic micronutrients like carotenoids, fat-soluble vitamins, and polyphenols (Iddir et al., 2021; Xavier & Mercadante, 2022). However, SHIME has a microbial fermentation compartment that allows the simulation of colonic digestion. This enables the study of microbiota-mediated transformations of dietary components (Singh et al., 2022; Zhu et al., 2024). While static models such as INFOGEST are highly suitable for standardized testing and initial research, dynamic models provide more detailed insights for regulatory assessments, product optimization, and advanced formulation research (Lesmes, 2023; Lopez-Rodulfo et al., 2025).

To illustrate these conceptual differences, several studies have reported quantitative variations in nutrient release and bioaccessibility under static and dynamic digestion conditions. One such example is carotenoid bioaccessibility, which increased by 182% when gastric lipase was incorporated into the INFOGEST static model, thereby indicating that even modest differences in enzyme composition can substantially influence the results (Iddir et al., 2021). Similarly, the TIM-1 model showed that polyphenol bioaccessibility in sugar sand by-products reached levels of over 100% which was more than two times higher than that of static model predictions due to the facilitated extraction and matrix breakdown by dynamic shear forces and pH control (Decabooter et al., 2023). Similarly, application of the SHIME model to evaluate selenium release from cooked rice showed a bioaccessibility range of 67–76% which pointed out the capacity of the model to simulate a realistic digestion scenario (Sun et al., 2017). These differences become more evident when specific food matrices and delivery systems are compared across model formats. (Massarioli et al., 2023) further demonstrated limitations of static digestion when studying bioaccessibility of peanut phenolics: p-coumaric acid reached 370% bioaccessibility and 127% transport efficiency across Caco-2 monolayers, whereas more complex p-coumaroyl derivatives showed only 7–100% bioaccessibility and 14–31% transport, indicating that static digestion fails to capture the dynamic transformation and release behavior of complex food matrices. In addition, (Cianciosi et al., 2020) found that the total phenolic content and antioxidant capacity of Manuka honey decreased significantly (*p* < 0.05) after static in vitro digestion, yet its biological activity in colon cancer cells remained partly preserved—highlighting that static models inadequately mimic dynamic intestinal interactions. (Song et al., 2023) reported that astragalosides from Radix Astragali undergo substantial biotransformation during small-intestinal digestion, with acetyl isomerization and deacetylation of astragaloside IV markedly increasing antioxidant activity; however, static digestion could only partially reproduce these conversion patterns due to the absence of dynamic residence-time effects. Likewise, (Tian et al., 2019) observed that lactoferrin-loaded liposomes retained 52.3 ± 6.3% entrapment during static gastric digestion but decreased to 28.5% during simulated intestinal digestion, whereas dynamic models in other studies showed more progressive structural disintegration and enzyme interaction.

**Fig. 1:**
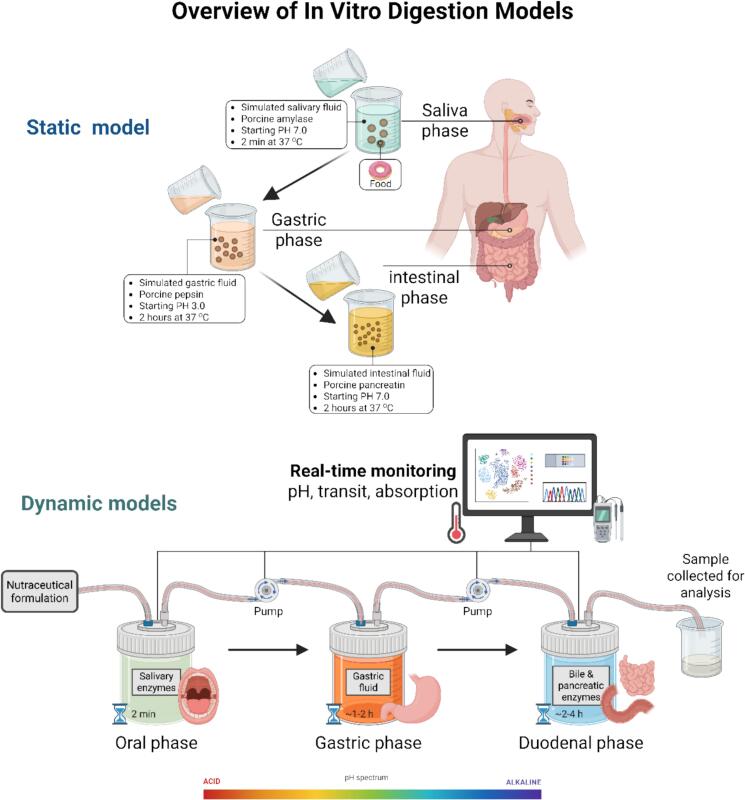
Schematic comparison between the static and dynamic in vitro digestion models. The static model depicts digestion in separate stages—oral, gastric, and intestinal—where each step is performed under controlled conditions with the use of standardized reagents (e.g., INFOGEST protocol). This model is the one that is the base for nutrient release and bioaccessibility assessment. Created in BioRender. Awlqadr, F. (2025) https://BioRender.com/z49rupb.

Despite these limitations, both static and dynamic models remain valuable for specific analytical, formulation, and translational applications in food research. Static models are most appropriate for the quick screening of food matrices, especially in high-throughput formats, and for comparing digestion outcomes in different processed foods. They have been utilized to evaluate the effect of encapsulation on antioxidant stability (Y.-L. Ma et al., 2024) and to assess the reduction of glycemic index in fiber-enriched functional bread (Subiria-Cueto et al., 2025). By contrast, time-dependent release and absorption, food-drug interactions, as well as testing probiotic viability and metabolite production over time, are among the areas where dynamic systems can be successfully applied (Araújo et al., 2025; Pinto et al., 2025). The coupling of these models with absorption systems such as Caco-2 monolayers or intestinal organoids further increases their predictive capacity (Llorens et al., 2025). However, dynamic models remain limited by high operational cost, technical complexity, and low accessibility, which reduce their practicality for routine use in food development laboratories. In addition, although they offer improved physiological realism, their predictive value still requires validation against human clinical outcomes (Brodkorb et al., 2019; Colombo et al., 2021). Accordingly, hybrid approaches combining static digestion with cellular assays or computational models are emerging to strike a balance among complexity, accessibility, and physiological relevance (Colombo et al., 2021; Le Feunteun et al., 2021). Table 1 gives a comparative summary of the in vitro digestion models which indicates the differences between the static and the dynamic systems. Static models based on the INFOGEST method are generally the first choice for harmonized bioaccessibility assay, while dynamic systems like TIM-1 and SHIME, which allow for factors such as pH gradients and microbial fermentation to be taken into account, are better at mimicking the physiological conditions. The use of hybrid models and omics-integrated approaches may improve predictions of nutrient release, absorption, and metabolism, while also facilitating applications beyond traditional boundaries, such as functional food design and micronutrient delivery. Fig. 1 defines the key differentials of static and dynamic in vitro digestion models. Overall, static models remain valuable for harmonized screening studies, whereas dynamic models provide greater physiological realism for advanced digestion research.

**Table 1:** Comparative Overview of Static and Dynamic In Vitro Digestion Models and Their Applications in Nutrient Bioaccessibility Studies.

Model Type	Model/System	Application Area	Key Findings/Outcomes	Reference
Static	INFOGEST	General nutrient digestion	Established a widely used harmonized protocol for in vitro digestion studies	(Minekus et al., 2014)
Static	INFOGEST	Bioaccessibility assays	Highlighted advances post-INFOGEST consensus	(Colombo et al., 2021)
Static	INFOGEST	Carotenoid bioaccessibility	Improved extraction and quantification from micelles	(Xavier & Mercadante, 2022)
Static	Modified INFOGEST	Carotenoid digestion with lipase	RGE lipase enhanced carotenoid bioaccessibility by 182%	(Iddir et al., 2021)
Static	Custom protocol	Functional bread matrix	Glycemic index reduced due to fiber-phenolic interactions	(Subiria-Cueto et al., 2025)
Static	INFOGEST + Caco-2	Nutraceutical assessment	Combined digestion and epithelial transport assessment of phenolic compounds	(Pinto et al., 2025)
Static	Multiple static models	Bioavailability simulation	Used to guide diet optimization strategies	(Rodrigues et al., 2022)
Static	Simulated digestion	Lactoferrin emulsion stability	Improved digestion stability and release behavior of the emulsion system	(Y.-L. Ma et al., 2024)
Static	Various (review)	Nutrient and bioactive release	Clarified harmonization needs in static protocols	(Alegría et al., 2015)
Static	Various	Bioaccessibility of food compounds	Extensive application in antioxidant release assays	(Lucas-González, Viuda-Martos, Álvarez, & Fernández-López, 2018)
Dynamic	TIM-1	Micronutrient digestion	Dynamic pH and enzyme flow improve realism of digestion	(Decabooter et al., 2023)
Dynamic	SHIME	Colonic fermentation	Microbiota-mediated nutrient conversion included	(Venema & Van den Abbeele, 2013)
Dynamic	SHIME	Selenium in rice	Bioaccessibility reached 76% in cooked rice	(Sun et al., 2017)
Dynamic	TIM-1	Maple syrup by-products	Polyphenol bioaccessibility exceeded 100%	(Decabooter et al., 2023)
Dynamic	INFOGEST + Caco-2	Mycotoxin-polyphenol interaction	Polyphenol presence influenced post-digestive transport behavior	(Llorens et al., 2025)
Dynamic	Various	Functional food validation	Supported design of safe infant formula and cereals	(Lesmes, 2023)
Dynamic	3D Food + digestion	Riboflavin delivery	Enhanced nanostructure stability and absorption	(Araújo et al., 2025)
Dynamic	Simulators + modeling	Micronutrient dynamics	Advocated hybrid modeling for real-time simulation	(Marze, 2017)
Dynamic	Omics-integrated models	System-wide food component digestion	Linked in vitro digestion to systems biology	(Capozzi & Bordoni, 2013)
Dynamic	Hybrid	Amino acid scoring	DIAAS model supported by digestion simulations	(Rutherfurd et al., 2015)

### 2.2 Compartmental simulation

The compartmental simulation of in vitro gastrointestinal digestion is an intricately designed stepwise method intended to imitate the physiological milieu of the human digestive tract, which includes the oral, gastric, intestinal, and colonic phases (Hur et al., 2011; Minekus et al., 2014). Each stage of the digestive tract involves specific enzymatic activity, pH, and changes in form due to the action of the digestive system.(Brodkorb et al., 2019) Such simulations are necessary when determining the bioaccessibility and stability of dietary nutrients, phytochemicals, and medicated compounds, particularly when considering the development of functional foods (Fernández-García et al., 2009; Nicolescu et al., 2023). The oral phase is usually short and can be identified by the mechanical destruction of food and enzymatic activity of salivary α-amylase at a pH close to neutral (6.8–7.2) (Minekus et al., 2014; Wickham et al., 2009). Besides initiating the hydrolysis of starch to maltose and dextrin, this stage mixes food with enzymes and starts the digestive processes (Freitas et al., 2018). As a result, the oral phase has a direct impact on the rest of the digestive process. Moreover, this phase is often neglected or simplified in many in vitro studies, which could lead to a significant underestimation of early enzymatic transformations (Dávila León et al., 2024).

**Fig. 2:**
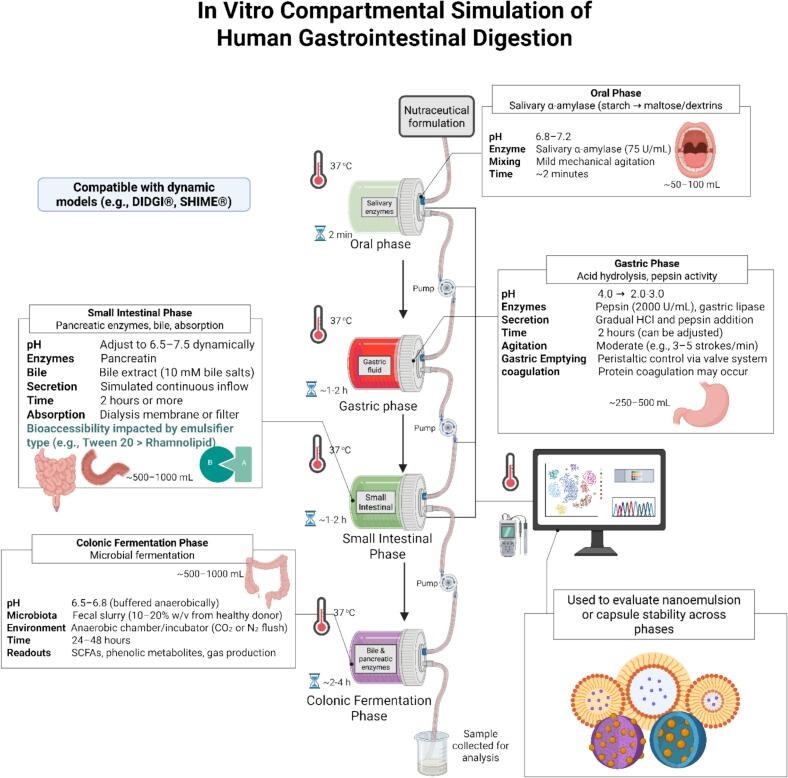
Compartmental simulation of gastrointestinal digestion using dynamic in vitro models. This illustration outlines the stepwise simulation of oral, gastric, small intestinal, and colonic digestion phases. Created in BioRender. Awlqadr, F. (2025) https://BioRender.com/834pdri.

The gastric phase simulates the acidic stomach environment (pH 2.0–3.0) and pepsin activity, leading to extensive protein hydrolysis (Kong & Singh, 2008; Minekus et al., 2014). Under these conditions, the stability and release behavior of delivery systems such as nanoparticles can also be affected (McClements, 2004; Salvia-Trujillo et al., 2013). Dynamic models such as DIDGI® can almost simulate gastric motility and enzyme kinetics better than static models, which result in better protein degradation for food matrices like casein gels (Logan et al., 2023). Moreover, acid-induced coagulation can also affect the release and solubility of encapsulated compounds, as demonstrated by the increased curcumin digestibility in lipid nanoparticles (Peng et al., 2018). The intestinal phase imitates the small intestine's milieu, generally with the help of pancreatin (a mixture of enzymes like trypsin, amylase, and lipase) and bile salts to recreate enzymatic hydrolysis and emulsification. Digestion was continued with the pH being 7.0–7.5, which also allowed the formation of micelles for lipids. The INFOGEST protocol is the most widely recognized harmonized approach for simulating this phase and has substantially improved comparability across laboratories, although interlaboratory variability can still arise from differences in enzyme activity, reagent preparation, and pH adjustment (Luz et al., 2024). In curcumin-loaded systems, bile salts and emulsifiers have caused a major change in the bioaccessibility of the compound, whereby Tween 20 was the best natural emulsifier, similar to rhamnolipid, for releasing curcumin in the intestinal phase (Yu et al., 2023). The addition of a colonic fermentation phase makes these simulations more relevant to the physiology, particularly for polyphenol-rich or fiber-rich foods. This phase involves fermentation of the applied sample by the gut microbiota of humans or their enzymatic extract (e.g., fecal slurry), which is usually done under anaerobic conditions at pH 6.8. The formation of short-chain fatty acids including acetate, propionate, and butyrate is the functional output. For instance, studies of gabiroba fruit powder indicated that antioxidants and polyphenols were stable even after colonic digestion, thus prebiotic capacity was implied (Foltz et al., 2021). This phase is mainly considered when evaluating non-absorbed dietary components and their transformation into beneficial metabolites. All phases have pH variation and ionic conditions that largely influence nutrient release, as demonstrated by Laloux et al. (2020), who found that AgNPs agglomerated in the acidic gastric phase but then disintegrated in intestinal fluids, which showed how the physicochemical properties of nanoparticles changed due to digestive dynamics (Laloux et al., 2020). In addition, pH adjustment during protein digestion affects the profile of released peptides and consequently their bioactivity potential as in the case of egg white protein digestion (Zhang et al., 2023). In summary, compartmental simulations provide important insight into nutrient bioaccessibility and transformation. Inclusion of oral, gastric, intestinal, and colonic phases provides a more complete picture of nutrient fate during digestion. Such models facilitate optimization of functional ingredients, encapsulated formulations, and nanocarrier systems thereby making it possible to link preclinical and clinical data for translational food and nutraceutical sciences. Table 2 provides a comparative description of the different in vitro model simulated digestion phases. Fig. 2 outlines advances made in vitro digestion phase modeling including integration of salivary, gastric, intestinal, and colonic environments. As outlined in Section 2.1, static and dynamic models differ primarily in how digestive conditions are applied, whereas compartmental simulation refers to the physiological phases represented within the model. In this section, the focus is on the compartmental organization of digestion rather than re-defining the operational distinction between static and dynamic systems (Daneshgar et al., 2024). This distinction helps link the structural organization of digestion phases with the broader methodological framework introduced earlier.

**Table 2:** Summary of Compartmental In Vitro Digestion Studies across Oral, Gastric, Intestinal, and Colonic Phases.

Simulated Phase	Model Type	Application	Key Outcome	References
Oral Phase	Static	Salivary amylase in initial digestion	Starch breakdown initiated, but often simplified	(Dávila León et al., 2024)
Gastric Phase	Dynamic (DIDGI®)	Protein digestion in casein gels	Improved breakdown under dynamic gastric conditions	(Logan et al., 2023)
Gastric + Intestinal Phase	Static	Curcumin-loaded nanoparticles	Tween 20 led to better bioaccessibility than natural emulsifiers	(Yu et al., 2023)
Colonic Fermentation	Static + Fecal slurry	Polyphenol fermentation from fruit powder	Stable antioxidant activity post-fermentation	(Foltz et al., 2021)
Full GIT Simulation	Static	Silver nanoparticle aggregation	pH-dependent transformation in gastric and intestinal fluids	(Laloux et al., 2020)
Gastric Phase	Static	Egg white protein digestion	pH affects peptide release and bioactivity	(Zhang et al., 2023)
All Phases	Static (INFOGEST)	Harmonized digestion protocol	Improved interlaboratory comparability, although variability may still occur	(Minekus et al., 2014)
All Phases	INFOGEST	Method harmonization	Supported broader harmonization of digestion procedures across laboratories	(Brodkorb et al., 2019)
Colonic Fermentation	Dynamic (SHIME)	Selenium bioaccessibility	Achieved 76% release in cooked rice	(Sun et al., 2017)
Colonic Fermentation	Dynamic (SHIME)	Microbiota simulation	Enables SCFA monitoring	(Venema & Van den Abbeele, 2013)
Multi-phase	Omics-integrated	System-wide food digestion	Metabolomics linked to digestion outcomes	(Capozzi & Bordoni, 2013)
Gastric + Intestinal	Dynamic (TIM-1)	Polyphenol release	Bioaccessibility exceeded 100% from food waste	(Decabooter et al., 2023)
All Phases	Dynamic	Infant formula & cereals	Dynamic validation of food safety and delivery	(Lesmes, 2023)
Intestinal Phase	Static	Lactoferrin emulsions	Improved nutrient stability	(Y.-L. Ma et al., 2024)
Small Intestine	Static	Fiber-fortified bread	Phenolic interaction reduced glycemic index	(Subiria-Cueto et al., 2025)
All Phases	Static	Nutrient screening	Supports diet design optimization	(Rodrigues et al., 2022)
Intestinal + Caco-2	Static	Polyphenol transport	Achieved >90% transport after digestion	(Pinto et al., 2025)
All Phases	Review	Protocol harmonization	Outlined limitations and improvements	(Alegría et al., 2015)
Intestinal Phase	Static + Caco-2	Mycotoxin–polyphenol interaction	Influenced compound uptake	(Llorens et al., 2025)
Small Intestine	Dynamic	Riboflavin from 3D foods	Enhanced nanostructure absorption	(Araújo et al., 2025)
All Phases	Hybrid	Simulators + kinetic models	Promoted real-time digestion mapping	(Marze, 2017)
All Phases	Static	Food nutrient availability	Discussed bioaccessibility of vitamins	(Vancoillie et al., 2024)
Stomach	TIM-1	Dynamic modeling	Improved representation of gastric pH control	(Minekus et al., 1995)
Small Intestine	Static	Polyphenol metabolism	Showed transformation during digestion	(Tagliazucchi et al., 2010)
Small Intestine	Hybrid	Protein digestibility scoring	Validated DIAAS system	(Rutherfurd et al., 2015)
Intestinal Phase	Static	Antioxidant release	Evaluated dietary antioxidant delivery	(Lucas-González, Viuda-Martos, Álvarez, & Fernández-López, 2018)
All Phases	Static	INFOGEST update	Summarized new developments	(Colombo et al., 2021)

### 2.3 Evolution of in vitro systems

The development of in vitro digestion (IVD) models has changed significantly. The transition has been from very simple first-generation static systems to elaborate and multi-compartmental platforms that are able to represent physiological and biochemical processes of the human gastrointestinal (GI) tract with higher fidelity. At a very rudimentary level, in vitro models recast the operation of the human digestive system using enzymes as catalysts in test tubes. While these models gave researchers insightful data on food breakdown, they still had limitations in terms of realism and reproducibility (Kiran et al., 2025). Initial methodologies rapidly singled out the last life phase due to the limited number of components addressed in the digestion, as well as lacking most of the physiological phenomena involved; whereas, the typical enzyme kinetics or pH-adjustments were being taken into account at the time. These were thus seen as the most basic of the physiological questions relating to the metabolic properties of the human body, even though on the market the models were at times the cheapest ones (Vancoillie et al., 2024).

One of the crucial stages in the development of static modeling was the standardization of the INFOGEST protocol as a consensus-driven digestion method. In accordance with such protocol, the defining characteristics of a generic digestion model are not only the reproducibility of the enzymatic activities, but also the clear specification of pH values and the neatness of the simulated transit of digests through the digestive system (Di Stasio et al., 2024). The second generation of the INFOGEST protocol (INFOGEST 2.0) improved the assignment of enzyme activities, the preparation of the matrix, and the structure of the food bolus. Consequently, it firmly established itself as the point of reference for studies on bioaccessibility, especially, in cases of polyphenols, proteins, and lipids (Brodkorb et al., 2019; Dávila León et al., 2024). Nevertheless, static models still have the disadvantages inherent to their nature, such as the absence of mechanical mixing and stationary enzyme concentrations, which do not exactly reflect the changing physiological environment (Chen et al., 2023). The subsequent evolution of the field focused less on redefining basic digestion phases and more on improving physiological realism, absorption integration, and compatibility with advanced analytical platforms. The TNO's TIM-1 (TNO Intestinal Model) is among the most well-known instances of such a dynamic digestion system capable of reproducing the movements that occur in the peristalsis, gastric emptying, secretion of enzymes and bile, and pH changes as they happen (Dupont et al., 2019). Accordingly, TIM-1 has been successfully applied in studies of drug release and nutrient accessibility, thereby setting conditions that are more in line with the physiological reality, however, it costs much more and is technically more complex (Havenaar & Bellmann, 2022; Liu et al., 2017). In the same way, SHIME (Simulator of the Human Intestinal Microbial Ecosystem) incorporates a colonic fermentation stage, which makes it an appropriate model for microbiome and prebiotic evaluation studies (Van de Wiele et al., 2015; Zhu et al., 2024). This transition has also included microfluidic gut-on-a-chip devices that enable real-time observation and simulation of epithelial cell reactions under strictly regulated fluid dynamics, fusing both the mechanical and cellular aspects of the reality (Ofori-Kwafo et al., 2025). Presently, such systems are being employed in nutrition and pharmaceutical studies to determine how drugs move through the body, assess their safety and check if they are metabolized in the right way with maximum precision. The monolayers of Caco-2 cells still hold the top position as the model that portrays the intestinal epithelial absorption most accurately, especially if in vitro digestion is used as a basis for the transport of lipophilic compounds, polyphenols, and bioactive peptides (Antal et al., 2024; Minekus et al., 2014; Ting et al., 2015). Dialysis tubing, while being less physiologically accurate, continues to be utilized as a method for simulating absorption by imitating the molecular weight cutoff barrier of the intestinal wall (Youhanna & Lauschke, 2021).

There are many examples of studies that used integrated systems, where in vitro digestion was combined with cell-based assays or simulated absorption techniques. One example is the study by (Nallathambi et al., 2020) who combined simulated gastrointestinal digestion with Caco-2 cell models to investigate intestinal transport of polyphenolic compounds, In contrast, Wang et al. (2025) merged TIM-1 with simulated colonic fermentation to evaluate the bioavailability of astaxanthin (Wang et al., 2025). Furthermore, the new hybrid platforms combine dialysis, enzymatic hydrolysis, and microbial fermentation, which represents complete GI simulation from the oral cavity to the colon, thus enabling the study of both host and microbial interactions with nutrients and contaminants (Laloux et al., 2020; Zhu et al., 2024). Recently, the publications have been concentrated on the significant role which dynamic models and absorption modules have in nanotechnology applications. It is shown that the change of nanoparticles in the gastrointestinal tract as well as their interaction with epithelial layers and mucosal barriers are factors that greatly influence bioavailability and safety (Laloux et al., 2020; Laloux et al., 2021). In addition, metabolomics and transcriptomics are being more and more used in these models for the sake of studying not only a compound's bioaccessibility but also its metabolic fate and the cellular responses (Ting et al., 2015; Zhan et al., 2024). The researchers have connected a number of compartments and have inserted physiological stimuli but, nevertheless, they have to encounter the problems. The existence of differences between various kinds of models, the absence of standardization for dynamic models, and the high costs of their operations have led to a restriction of their accessibility (Brodkorb et al., 2019). Still, the integration of static or dynamic digestion with advanced cell culture, microfluidics, and omics platforms offers significant potential for the predictive, high-throughput screening of nutrient bioavailability as well as risk assessment, especially when it is synergized with EFSA and FDA regulatory frameworks (Radio et al., 2024; Wang et al., 2025). Table 3 delineates the generational evolution of in vitro digestion systems from simple static models to dynamic and bioengineered platforms.

**Table 3:** The development of in vitro digestion systems and their integration with absorption platforms.

Generation	Model/System	Key Features	Absorption Integration	Representative References
First-gen static	Simple beaker assays	Separate gastric or intestinal phases, fixed conditions	None	(Vancoillie et al., 2024)
Multi-phase static	INFOGEST protocol	Sequential oral, gastric, and intestinal steps with fixed pH and enzyme conditions	None or dialysis assays	(Brodkorb et al., 2019; Minekus et al., 2014)
Dynamic models	TIM-1, TIM-2, DIDGI	Controlled transit, enzyme secretion, dialysis filtration	Integrated dialysis recovery	(Liu et al., 2017; Ting et al., 2015; Verwei et al., 2006)
Coupled absorption	INFOGEST + Caco-2	Digestion + transport through epithelial cells	Caco-2-based intestinal transport evaluation	(Xu et al., 2022)
Next-gen bioengineered	Microfluidic digestion chips; gut-on-a-chip	Dynamic digestion, microflow, mechanical stimulation	Caco-2 or primary cells in channel	(Zhan et al., 2024)

## 3 Analytical techniques for characterizing digestion products

### 3.1 Nutrient and bioactive compound quantification

**Fig. 3:**
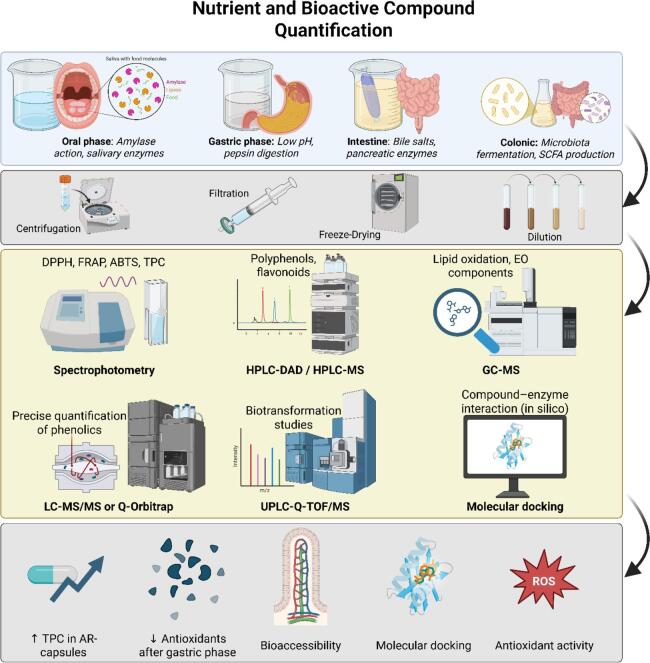
Workflow and analytical techniques for evaluating bioaccessibility and transformation of food bioactives during in vitro digestion. Created in BioRender. Awlqadr, F. (2025) https://BioRender.com/08qetk3.

To evaluate the health-promoting effects of food products, it is crucial to measure not only the nutrients but also the bioactive compounds released during digestion. Multiple advanced analytical techniques, such as spectrophotometry, high-performance liquid chromatography (HPLC), gas chromatography–mass spectrometry (GC–MS), and liquid chromatography-tandem mass spectrometry (LC-MS/MS), are broadly used to check the release and stability of these molecules in vitro digestion (Donno et al., 2020). Among these, spectrophotometry is still considered the core method for evaluating total phenolic content (TPC), total flavonoid content, and antioxidant capacity (e.g., DPPH, FRAP, ABTS) (Donno et al., 2020). In addition to conventional spectrophotometric cuvette-based measurements, microplate readers are widely used for the high-throughput quantification of antioxidant activity, including DPPH, FRAP, and ABTS assays. Microplate-based detection significantly increases analytical efficiency by reducing sample volume, minimizing reagent consumption, and enabling the simultaneous measurement of dozens of samples under identical conditions. The 96-well format also improves reproducibility by controlling the incubation times and temperature more uniformly and allowing automated mixing, kinetic monitoring, and multi-wavelength scanning (Arsul et al., 2022). Recent studies have demonstrated that microplate readers provide antioxidant activity values comparable to traditional spectrophotometers, while offering higher sensitivity and throughput—making them especially suitable for digestion studies where numerous fractions must be analyzed across different phases (Arsul et al., 2022). Therefore, microplate reader platforms represent an important extension of classical antioxidant assays and have become essential tools for evaluating the stability of bioactive compounds during in vitro gastrointestinal digestion. Overall, spectrophotometric methods remain useful for rapid screening of antioxidant-related changes during digestion, but chromatographic and mass spectrometric platforms provide substantially greater resolution for identifying structural transformation products and phase-specific bioaccessibility changes. Across recent studies, LC-based methods were particularly valuable for tracking digestion-induced modification of phenolics and other bioactives, whereas sample preparation procedures such as fractionation and extraction critically influenced detection sensitivity and analytical reliability. GC–MS is more appropriate for volatile compounds or derivatized metabolites (Castaldo et al., 2021). However, it has been used in polyphenol digestion to identify lipid oxidation products and volatile components released during digestion, as described by Forbes-Tretola et al. (2023) in digested honey and berry matrix studies. The combined use of in vitro digestion scenarios such as INFOGEST with complementary analytical platforms enables more robust multimodal assessment of digestion products (Tretola et al., 2023). Recent studies further show that combining chromatographic platforms with antioxidant assays and in silico tools improves interpretation of digestion-induced structural change and potential bioactivity. In particular, integrated LC-based analysis, bioactivity assays, and molecular docking approaches have strengthened mechanistic understanding of compound transformation, retention of antioxidant potential, and possible absorption-related interactions during digestion (Gkountenoudi-Eskitzi et al., 2023; Shahidi & Dissanayaka, 2023; Song et al., 2023). Appropriate sample preparation, including freeze-drying, solid-phase extraction (SPE), and centrifugation, remains crucial before analytical assessment. For instance, Giampieri et al. (2018) highlighted that post-digestive fractionation increases the detection sensitivity for the main antioxidants and avoids the occurrence of interference from digestive enzymes or bile salts (Giampieri et al., 2018). These and other techniques provide complementary advantages that are essential for monitoring the stability, transformation, and potential bioavailability of nutrients and bioactive compounds under simulated gastrointestinal conditions (Dima et al., 2024). Improvements in hyphenated techniques (e.g., LC-MS combined with HRMS or GC–MS with thermal desorption) continue to enhance sensitivity and compound identification, thereby providing important analytical support for functional food development, nutraceutical design, and pharmacokinetic evaluation (Dima et al., 2024). These analytical platforms are essential for supporting formulation strategies aimed at preserving and monitoring health-related compounds during digestion and absorption. Table 4 summarizes the principal analytical techniques used to characterize digestion products. Fig. 3 shows a schematic representation of the in vitro digestion experimental setup, depicting the digestion stages, sample processing, and advanced analytical techniques such as LC-MS/MS, GC–MS, and molecular docking.

**Table 4:** Summary of analytical techniques employed to characterize digestion products.

Study	Analytical Technique	Main Focus
(Song et al., 2023)	UPLC-Q-TOF/MS	Transformation of astragalosides and flavonoids during simulated gastrointestinal digestion
(Cianciosi et al., 2020)	HPLC-ESI-MS/MS plus bioactivity assays	Polyphenol stability and post-digestive bioactivity of Manuka honey
(Gkountenoudi-Eskitzi et al., 2023)	HPLC-DAD, HPLC-DAD-ESI-MS	Phenolic profiling and post-digestive characterization of acorn- and chickpea-fortified breads
(Duque-Soto et al., 2022)	HPLC-ESI-TOF-MS	Gastrointestinal stability and bioaccessibility of olive leaf polyphenols
(Castaldo et al., 2021)	UHPLC-Q-Orbitrap HRMS	Post-digestive profiling and bioaccessibility assessment of fennel waste polyphenols
(Gómez-Mejía et al., 2022)	HPLC-DAD, HPLC-MS/MS	Bioaccessibility of tea polyphenols and caffeine during simulated gastrointestinal digestion
(Cárdenas-Castro et al., 2021)	GC/MS	Volatile compound formation during in vitro digestion of tomato-based beverages
(Sánchez-Velázquez et al., 2021)	UHPLC and antioxidant assays (DPPH, ABTS)	Stability, bioaccessibility, and antioxidant activity of blackberry polyphenols during simulated gastrointestinal digestion
(Değirmencioğlu et al., 2016)	Spectrophotometry (DPPH, ABTS)	Changes in antioxidant activity during simulated gastrointestinal digestion
(Pereira-Caro et al., 2021)	Spectrophotometry (DPPH, ABTS)	Antioxidant-related changes during simulated gastrointestinal digestion
(Peña-Vázquez et al., 2022)	HPLC-ESI-MS/MS plus antioxidant assays	Post-digestive polyphenol changes and antioxidant-related effects in sweet orange
(Lee et al., 2020)	LC-MS	Changes in quercetin and resveratrol during simulated human digestion
(Papillo et al., 2019)	HPLC	Degradation and bioaccessibility of curcuminoids during gastrointestinal digestion
(Sagratini et al., 2013)	LC-MS	Soyasaponin content and bioaccessibility in legumes after simulated digestion
(Van de Velde et al., 2018)	HPLC	Bioaccessibility of anthocyanins and ellagitannins from blackberry during digestion and fermentation
(Kamal et al., 2023)	HPLC	Release and bioaccessibility of phenolic compounds from date palm fruits
(Zagórska et al., 2023)	Spectrophotometry, HPLC	Bioaccessibility of ginger bioactive compounds during simulated digestion
(Hashemi et al., 2023)	HPLC	In vitro digestion-related stability and intestinal absorption of curcumin-loaded zein nanoparticles

### 3.2 Structural and molecular characterization

Structural and molecular characterization methods are central to the application of advanced analytical techniques in food research. Instruments such as NMR, FTIR, and different microscopy tools (SEM, TEM, CLSM) have proven essential for measuring food matrix disintegration and the fate of nutrients during in vitro digestion. These techniques not only reveal structural changes in food matrices, but also improve understanding of the molecular interactions, bioaccessibility, and release dynamics of bioactive compounds and nutrients. NMR spectroscopy is the main method of choice for describing molecular interactions, changes in the structure of molecules, and the transformation of metabolites through digestion. Recent NMR-based studies have shown that this technique is particularly useful for characterizing noncovalent interactions between food bioactives and biological macromolecules during digestion. For example, ^1^H NMR and DOSY NMR have been used to identify polyphenol interactions with mucin and salivary proteins during the oral phase, with spectral shifts indicating tightly bound complexes that may influence bioaccessibility (Ciampa et al., 2022).

FTIR spectroscopy is one of the methods that can uncover changes in the structure and the characteristics of substances during digestion, especially for proteins, polysaccharides, and lipids. Across recent studies, FTIR has been widely used to monitor structural changes in proteins, polysaccharides, and lipid-containing systems during digestion. ATR-FTIR has been applied to detect changes in starch–lipid complexes through shifts in the amide I and II regions, while FTIR spectral deconvolution has also revealed protein structural transitions, such as α-helix-to-β-sheet conversion, associated with increased digestibility (Zhao et al., 2022). In addition, because FTIR does not require sample destruction or labeling, it is particularly suitable for emulsions, encapsulates, and hydrocolloid matrices (Kong & Yu, 2007). Sharif et al. (2022) reported that TEM was used to observe nanostructure changes of lipid-based nanoemulsions during the duodenal digestion that included micelle transformation and bile salt interactions. Microscopy-based techniques, particularly TEM and CLSM, provide complementary visualization of digestion-induced microstructural changes. TEM has been used to observe nanostructural transformations in lipid-based nanoemulsions during duodenal digestion, including micelle formation and bile salt interactions. CLSM is similarly valuable for real-time observation of multiphase systems, as fluorescence staining enables localization of bioactive compounds, lipid droplets, and protein aggregates during digestion (Sharif et al., 2020). Studies using CLSM further showed that proteolysis disrupts emulsified casein structures and promotes formation of lipid–protein assemblies during the intestinal phase, while the technique also supports monitoring of emulsion stability and release behavior in complex digestion chambers (Mun et al., 2017; Velderrain-Rodríguez et al., 2019). This mode of combining techniques is generally utilized for the in-depth structural metabolic profiling. Integrated analytical strategies further strengthen structural interpretation during digestion. For example, combined FTIR, CLSM, and SEM analysis has been used to characterize curcumin-loaded nanoparticles across oral-to-intestinal digestion phases, revealing structural swelling, partial matrix disintegration, and reduced fluorescence intensity associated with lower antioxidant retention (Peng et al., 2018). Similar multi-technique approaches have also been applied to insect protein isolates and bioengineered protein–polysaccharide conjugates, where SEM, FTIR, and TEM collectively helped characterize protein breakdown, amide-bond changes, and pH-dependent structural stability in gastrointestinal environments (Adugodi, 2022; Ozel et al., 2020). Together, these structural and molecular characterization methods provide mechanistic insight into not only the extent of compound release, but also the ways in which bioactives move, transform, and interact with other components in the digestive environment. Their incorporation into in vitro digestion protocols improves understanding of formulation strategies for functional foods, encapsulation systems, and nutraceutical delivery platforms. Fig. S1 summarizes the structural and molecular characterization methods commonly used in in vitro digestion research, and Table 5 outlines representative applications of these techniques in digestion studies.

**Table 5:** Structural and Molecular Characterization Techniques in Digestion Studies.

Study (Author, Year)	Technique(s)	Main Focus
(Zhang et al., 2021)	NMR	Interaction of polyphenols with mucin and salivary proteins during oral digestion
(Kleekayai et al., 2021)	High-resolution NMR	Monitoring bioactive peptide release from milk proteins
(Raza et al., 2022)	FTIR	Structural changes in starch-lipid complexes under digestion
(Chávez-Murillo et al., 2018)	ATR-FTIR	Protein structural transitions indicating digestibility
(Bayrak et al., 2021)	SEM	Pore expansion and surface erosion in digested pea protein matrix
(Roger et al., 2023)	TEM	Micelle transformation in lipid-based nanoemulsions during digestion
(Cheng et al., 2022)	CLSM	Disruption of emulsified casein and visualization of protein-lipid interaction
(Zhang et al., 2022)	CLSM	Tracking lipid droplet breakdown in emulsions under digestion
(Carpenter et al., 2019)	FTIR, SEM, CLSM	Encapsulated curcumin structural changes through digestion
(Adugodi, 2022)	SEM, FTIR	Proteolytic digestion of insect protein isolate
(Han et al., 2017)	FTIR	Structural resilience of Maillard protein-polysaccharide conjugates
(Yang et al., 2019)	FTIR	Starch and protein matrix interactions in gastric digestion
(Wekre et al., 2019)	NMR, CLSM	Bioaccessibility of polyphenols from digested algae matrix
(Liang et al., 2018)	SEM	Surface morphology changes in digested cereal fiber
(Yang et al., 2020)	CLSM	Monitoring lipid oxidation during digestion using fluorescence
(Kim et al., 2023)	FTIR, NMR	Protein–polyphenol binding and breakdown dynamics
(Zhuang et al., 2015)	SEM	Microstructural degradation of gelatin during enzymatic digestion
(Naso et al., 2019)	TEM	Nanoemulsion interaction with bile salts
(Contardo et al., 2025)	FTIR	Conformational changes in chickpea protein isolates
(Florczuk et al., 2023)	CLSM	Visualizing antioxidant localization in fermented dairy matrices
(Constantin et al., 2021)	NMR	Metabolic fingerprinting of digestive fluids with encapsulated anthocyanins
(Kansandee et al., 2024)	FTIR	Gastrointestinal simulation of prebiotic polysaccharide conformation
(Bisht et al., 2021)	NMR	Metabolite release from digested plant lipids

### 3.3 OMICS technologies in digestion studies

OMICS-based approaches, including metabolomics, proteomics, and lipidomics, have substantially expanded the analytical scope of in vitro digestion research. These techniques enable systems-level characterization of small molecules, peptides, and lipid intermediates generated during digestion, thereby providing mechanistic insight beyond that achievable with conventional biochemical assays. Metabolomics is increasingly used to profile digestion-derived metabolites across different phases, proteomics helps identify bioactive and digestion-resistant peptide sequences, and lipidomics provides detailed information on lipolysis, lipid remodeling, and intermediate lipid species. Across recent studies, these platforms have been applied to plant, dairy, meat, legume, and emulsion-based systems to clarify matrix-dependent nutrient transformation, bioactive peptide release, and digestion-associated metabolic shifts (Ajayi et al., 2023; Dávila León et al., 2024; M. Li et al., 2024; Hu et al., 2023; Senadheera et al., 2022; Tang et al., 2022; Z. Ma et al., 2024). Bioinformatics and systems biology tools further support interpretation of these datasets by enabling pathway mapping, interaction analysis, and predictive modeling of digestion-related molecular behavior (Perez De Souza et al., 2020; Malvar et al., 2022; Sadeghi Ekbatan et al., 2018). However, methodological heterogeneity across digestion models, food matrices, metabolite libraries, and bioinformatic pipelines continues to limit cross-study comparability and remains a major barrier to standardization (Rasera et al., 2023). Taken together, these findings indicate that OMICS approaches strengthen mechanistic interpretation of nutrient transformation during digestion, although greater harmonization is still required before routine comparative application across studies. **Fig. S2** illustrates the integration of metabolomics, proteomics, and lipidomics with in vitro digestion systems**.**

## 4 Applications in nutrient bioaccessibility and bioavailability

A clear distinction between nutrient bioaccessibility and bioavailability is essential in food science and nutrition. Bioaccessibility refers to the fraction of a compound released from the food matrix during digestion and made available for absorption, whereas bioavailability refers to the absorbed and physiologically active fraction that reaches systemic circulation (Grundy et al., 2024; Rodrigues et al., 2022). This distinction is particularly important when in vitro models are used to measure the effectiveness of nutrients and bioactive compounds. **Fig. S4** illustrates the key differences between bioaccessibility and bioavailability, together with the main in vitro digestion models, influencing factors, and encapsulation strategies. Across studies, in vitro digestion models have consistently shown that bioaccessibility does not necessarily reflect true absorption, highlighting the importance of distinguishing between these two concepts in analytical interpretation (Hayes et al., 2020). These models are widely used to evaluate macronutrient digestion, with static systems such as INFOGEST providing standardized simulation of starch hydrolysis, proteolysis, and lipid emulsification, whereas dynamic platforms such as TIM-1 and SHIME better reflect physiological digestion kinetics and stepwise lipid release (Brodkorb et al., 2019; Oosterveld et al., 2016). Across recent studies, processing conditions and matrix structure have emerged as key determinants of macronutrient digestion, with thermal treatment, mechanical shear, starch retrogradation, and high-pressure processing altering glucose release and protein digestibility to different extents (Lian et al., 2024; Mulla et al., 2022). Gastric pH remains another major variable because enzyme activity, particularly pepsin function, is highly sensitive to acidic conditions and therefore strongly influences protein cleavage efficiency (Srivastava et al., 2018). Studies on micronutrients and bioactive compounds have been substantially supported by in vitro digestion models. Calcium, iron, vitamins, and polyphenols have been widely examined using in vitro digestion systems to evaluate release, solubility, stability, and transformation. Across recent studies, calcium and iron bioaccessibility were strongly influenced by the surrounding physicochemical environment, including pH, ionic conditions, fiber inclusion, and reducing agents such as ascorbic acid, all of which affected solubility and intestinal availability (Sabatier et al., 2020; Whisner & Weaver, 2017). Similarly, lipid-based delivery systems improved the digestion stability and bioaccessibility of vitamins such as vitamin D, while the fate of polyphenols remained highly matrix-dependent and was strongly shaped by digestive pH, enzymatic interactions, and binding to other food components (Karademir et al., 2024; Tan et al., 2021). In some cases, digestive degradation did not eliminate biological functionality, as shown by chlorogenic acid transformation into caffeic acid derivatives that retained antioxidant-related activity after intestinal digestion (Dawidowicz & Typek, 2017). Modern nutrient delivery systems such as encapsulation, nanoemulsions, and solid lipid nanoparticles have significantly enhanced nutrient bioaccessibility. Recent studies consistently indicate that encapsulation and lipid-based delivery systems enhance the bioaccessibility and digestion stability of bioactive compounds, particularly hydrophobic nutraceuticals. pH-responsive nanoparticle systems, alginate–chitosan beads, γ-cyclodextrin metal–organic framework–micelle combinations, and food-grade emulsification strategies have all been shown to improve compound protection, micellarization, and intestinal availability during digestion (Gong et al., 2025; Henao-Ardila et al., 2024; Oh et al., 2025; Silva et al., 2021). Similar trends have also been reported for vitamin-loaded zein nanoparticles, which improved vitamin stability during simulated gastric conditions (Karoshi et al., 2024).

Functional food development has expanded the application of in vitro digestion models for predicting post-digestive performance. Across functional food studies, in vitro digestion models have been increasingly used to compare formulation performance and predict post-digestive efficacy. A recurring trend is that encapsulated or nanoformulated systems improve the retention, uptake, and biological activity of compounds such as curcumin and omega-3 fatty acids relative to their non-protected forms, while combined INFOGEST–Caco-2 systems provide stronger insight into intestinal transport and cellular uptake (Islam et al., 2018; Ryu et al., 2024; Shah et al., 2016; Wang, Gong, & Zhou, 2025). Similar formulation-dependent effects have also been reported for probiotics, where encapsulation improves survival under gastric and bile stress conditions (Li et al., 2016). These findings are important for linking in vitro predictions with in vivo outcomes of functional food development. Recent studies have also integrated bioinformatics and OMICS analyses with in vitro digestion to examine nutrient transformation at the molecular level. For example, metabolomic profiling of digested milk peptides identified newly formed antihypertensive tripeptides such as VPP and IPP, illustrating how systems-level approaches can strengthen mechanistic interpretation and improve the predictive value of digestion models for nutraceutical innovation (Rutella et al., 2016). Overall, these applications demonstrate the value of in vitro digestion models for advancing understanding of nutrient bioaccessibility and bioavailability across both macro- and micronutrient categories. Their use continues to inform the development of evidence-based functional foods and supplements, as summarized in Table S3.

## 5 Recent innovations and hybrid approaches

### 5.1 Integration with cell culture systems

The use of in vitro digestion models with intestinal epithelial cell culture systems, such as Caco-2 and HT29-MTX, is a necessary step to determine the absorption, permeability, and transport mechanisms of nutrients, bioactive compounds, and pharmaceutical agents (Antal et al., 2024). These coculture or monoculture models aid in mimicking the human intestinal environment, thus enabling scientists to judge how compounds behave after digestion in biologically relevant systems (Vitale et al., 2025). Caco-2 derived from the human colon adenocarcinoma cells are widely used because they have the ability to automatically differentiate into enterocyte-like cells with tight junctions and microvilli that resemble the small intestinal epithelium (Zou et al., 2024). Hernandez-Patlan et al. (2018) employed Caco-2 cells to estimate curcumin (CUR) solubility and permeability. Their research revealed that curcumin complexed with polyvinylpyrrolidone (PVP) remarkably elevated its permeability through Caco-2 monolayers as compared with free curcumin indicating that excipients can quicken intestinal transport by facilitating solubility and cellular uptake (Hernandez-Patlan et al., 2018). Zhou et al. (2018) calculated the percentages of absorption of phenylethanoid glycosides (PhGs) like salidroside and acteoside by using the Caco-2 model. They found that saccharide diffused passively through the membrane, whereas the low Papp values (4.75 × 10^−7^ cm/s) of acteoside suggested that the substance was affected by P-glycoprotein (P-gp)-mediated efflux, thus, the absorption was limited (Zhou et al., 2018). This is an illustrative instance of employing Caco-2 cells for the revelation of efflux transport barriers for natural compounds (Zhou et al., 2018). Fernandes et al. (2021) demonstrated that the bioaccessibility and uptake of chlorophyll pigments from *Scenedesmus obliquus* depended on food matrix with the highest uptake in Caco-2 cells for the isolated chlorophyll extract (e.g., hydroxypheophytin a: 102.53%) while the absorption dynamics were determined by matrix composition (Fernandes et al., 2021). The HT29-MTX cell line, derived from mucus-secreting goblet cells, is generally used together with Caco-2 cells as a model of the human intestinal epithelium covered by mucus. The co-culture system revealed a more precise prediction of the in vivo permeability of mucosally absorbed compounds. To illustrate, in vitro digestion products of green tea catechins showed a dramatically higher epithelial uptake, when co-administered with flavonol-rich foods such as onion peel and Dendropanax morbifera, suggesting that dietary matrices regulate the availability of the compound (Choi et al., 2017). According to Liu et al. (2022), gut microbiota and Caco-2 cells could serve as a model to study the effects of dietary components (e.g., grapes) on intestinal absorption of pesticides, such as triadimefon. Their data reconfirmed that gut microbiota decreased triadimefon bioaccessibility and transepithelial transport across Caco-2 monolayers by as much as 32.81% (Liu et al., 2022). Moreover, Gille et al. (2019) reported that sonication of *Phaeodactylum tricornutum* improves carotenoid bioaccessibility and absorption. Among other carotenoids, the uptake of fucoxanthin in Caco-2 cells was the most significant thus illustrating the function of cell models in determining the influence of processing on nutrient availability (Gille et al., 2019). These studies together confirmed that Caco-2 and HT29-MTX cell culture systems are fundamental tools in mechanistic absorption studies. They open the windows to both passive and active transport routes. Transport proteins, for example P-gp, are of particular interest. Moreover, the impact of the food matrix and processing on the absorption is studied (Iftikhar et al., 2020). The absorption changes upon the administration of co-administered compounds or due to the microbiota (Kim, 2023). The addition of the triple co-culture system (Caco-2 + HT29-MTX + Raji B) is considered an improvement that could more accurately simulate the M-cell environment for the transport of particles or microbes (Vincentini et al., 2022). As these methods proceed, the combination of organ-on-chip and 3D intestinal models with the classic Caco-2/HT29-MTX systems will deepen the physiological relevance of nutrient absorption models (Pimenta et al., 2022).

### 5.2 3D bioprinted gut models and organ-on-chip

Several developments in in vitro digestion technologies have been brought about by recent advancements, leading to their wider application in the assessment of nutrient release, physiological absorption, and even giving a mechanistic insight into the gastrointestinal (GI) uptake (Lucas-González, Viuda-Martos, Pérez-Alvarez, & Fernández-López, 2018). Among all of them, 3D bioprinted gut models and organ-on-chip systems have gained a significant amount of attention, are increasingly recognized as advanced platforms for representing the complex structure and function of the human intestine with higher physiological fidelity for nutrient absorption and bioavailability of compound studies (Song et al., 2025). Recent advances in microphysiological systems (MPS) have greatly strengthened the physiological relevance of in vitro gastrointestinal digestion research by integrating 3D bioprinting, organoids, and microfluidic gut-on-chip technologies. According to (Mathur et al., 2025), MPS platforms recreate intestinal anatomy, digestion-associated mechanical forces, mucus secretion, and microbial interactions far more realistically than static INFOGEST assays, overcoming long-recognized limitations related to pH stability, shear stress, and epithelial complexity. In these systems, intestinal organoids and 3D-bioprinted tissues maintain native cellular architecture, including villus-crypt gradients, enabling improved modeling of nutrient absorption and epithelial barrier responses. (Song et al., 2025) demonstrated that a coaxial-bioprinted tubular small-intestine chip incorporating multiple human intestinal cell types produced functional epithelial polarization, physiologically relevant gene expression patterns, and structural fidelity superior to 2D Caco-2 monolayers, making it highly suitable for digestion and nutrient-transport studies. Microfluidic gut-on-chip platforms further enhance physiological accuracy. (Thomas et al., 2023) detailed how controlled fluid flow, peristalsis-like cyclic strain, and co-culture with gut microbiota enable dynamic simulation of digestion-related shear forces and real-time nutrient transport, improving the predictive accuracy of food-component absorption assessments compared with traditional models. Similarly, advanced human intestine-on-a-chip systems described by (Macedo et al., 2023) recreate epithelial–stromal and epithelial–endothelial interfaces under continuous luminal flow, allowing mechanistic evaluation of digestion, barrier integrity, and personalized nutrient responses that are unattainable with conventional gastrointestinal simulators. Collectively, these studies suggest that 3D bioprinted intestinal tissues and gut-on-chip platforms provide a more realistic mechanical, biochemical, and cellular environment for studying digestion and absorption, addressing critical limitations of static in vitro digestion and significantly improving in vitro–in vivo correlation. The conventional static and dynamic models are insufficient to recreate the dynamic microenvironment and tissue-level architecture of the intestinal epithelium. So as to settle this, the researchers have started applying the 3D bioprinting technologies in the production of gut tissue structures made of enterocytes, goblet cells, and immune cells surrounded by a hydrogel-based scaffold, which represents the multilayered anatomy of the intestinal wall. For example, Chen et al. (2023) and Rudolph et al. (2022) could bioprint villus-like architectures with Caco-2 and HT29-MTX cells that not only combine the absorptive and mucus-secreting properties but also show better permeability and tight junction integrity when compared to 2D monolayers. The organ-on-chip (OoC) platforms equipped with microfluidics connected to cell culture chambers aim to simulate the dynamic flow, peristalsis, and nutrient gradients for better physiological relevance (Chen et al., 2023; Rudolph et al., 2022). Palamà et al. (2025) created a gut-on-chip system with motion similar to peristalsis and co-cultured human intestinal epithelial cells under flow conditions to achieve morphology that is differentiated, mucus secretion, and nutrient transport, thus mimicking in vivo responses (Palamà et al., 2025). Such models allow the monitoring of nutrient absorption and microbial-host interactions over time under controlled shear stress and oxygen gradients, which is not possible with conventional models (Hewes et al., 2020). Organ-on-chip devices have come out as a tool with an improved prediction of nutrient bioavailability and compound metabolism, especially for lipophilic bioactive compounds, such as curcumin, omega-3 fatty acids, and polyphenols. Faralli et al. (2019) used this platform to investigate curcumin nanoemulsions and the results showed a markedly increased apparent permeability and transepithelial transport which were consistent with the in vivo pharmacokinetic profiles (Faralli et al., 2019). Additionally, the integration of hepatic and gut modules in multiorgan chips gives the possibility of simulating first-pass metabolism and thus, considerably before in vivo, it advances the field of nutraceutical testing (Fernandes et al., 2021).

One of the most important aspects of these next-generation models is that they can be combined with real-time imaging, multi-omics readouts (transcriptomics and proteomics), and machine learning-based algorithms for predictive modeling. An example is the transcriptomics profiling by Zhang et al. (2024) applied to a gut-chip exposed to digested spinach-derived polyphenols, which shows the regulation of oxidative stress pathways and tight junction gene expression—the same insights that traditional assays cannot offer (Zhang et al., 2024). These improvements come with problems such as high cost, scalability, and technical difficulty in long-term coculture and microfluidic integration. Nevertheless, bioprinted and chip-based gut models are progressively being perfected and are likely to become increasingly useful for evaluating dietary interventions, drug delivery systems, and host–microbe interactions, although broader adoption will depend on improved standardization, scalability, and cost-effectiveness. **Fig. S5** depicts the transition from traditional digestion models to advanced three-dimensional bioprinted gut and gut-on-chip platforms. These systems utilize hydrogel scaffolds, real-time imaging, and integrated omics/AI tools to simulate peristalsis, nutrient gradients, and epithelial interactions, significantly improving the physiological relevance of absorption studies.

### 5.3 AI and computational models

Artificial intelligence (AI) and computational modeling are increasingly being explored in in vitro digestion research to support prediction of nutrient release, transformation, absorption, toxicity, and metabolic fate under simulated gastrointestinal conditions. These approaches may integrate physicochemical properties, enzyme activity profiles, pH dynamics, digestion kinetics, and OMICS-related datasets to model digestive responses across different food matrices and physiological settings. Machine-learning and deep-learning methods have shown early promise in estimating intestinal permeability and bioaccessibility of peptides and polyphenols, although their performance remains dependent on dataset quality, model design, and external validation (Elumalai & Muthuvel, 2021; Li et al., 2022; Wang et al., 2023). Computational fluid dynamics, finite element analysis, and molecular simulations may also improve mechanistic interpretation by modeling enzyme diffusion, shear stress, nutrient partitioning, and controlled release behavior under digestion-relevant conditions (Bastos et al., 2020; Matsumura et al., 2020). In addition, integration of dietary, clinical, and microbiome data may help translate in vitro digestion outputs into more personalized predictions of digestive and metabolic responses; however, such applications remain at an early stage and have not yet been widely validated in food digestion models (Johnson et al., 2019; Pan et al., 2024; Zeevi et al., 2015) Overall, although AI-based tools may support data integration and predictive modeling, their practical application in validated digestion systems remains limited and requires further study before routine adoption in food chemistry research (Son, 2022).

## 6 Challenges and limitations

Despite significant progress in in vitro digestion modeling, several technical and methodological limitations persist, constraining its translational accuracy and applicability in nutrition and food science. A critical challenge is the lack of standardized protocols across laboratories and research institutions. Although efforts such as the INFOGEST consensus have provided valuable frameworks, variability in enzyme sources, enzyme activities, incubation times, and buffer compositions continues to affect the comparability of the results across studies. For example, even within the widely used INFOGEST model, differences in the source and activity of pancreatin or bile salts can lead to divergent outcomes in bioaccessibility values, limiting the reproducibility and consensus across datasets. Another major limitation is the incomplete simulation of the gut microbiota, particularly in the colon. Most static models simulate the oral, gastric, and small intestinal phases, often neglecting the complex metabolic and fermentative activities of the colonic microbiota, which are vital for the transformation of polyphenols, fibers, and resistant starches. Although some dynamic models, such as SHIME and TIM-2, attempt to incorporate microbial communities, they require complex operations, high costs, and maintenance of strict anaerobic conditions, which restrict their widespread adoption. Consequently, in vitro studies may underestimate the extent of metabolite bioconversion and the microbial-host interactions that occur in the colon. Furthermore, there is often a poor correlation between in vitro and in vivo data, especially when models fail to consider physiological factors such as peristalsis, hormonal feedback, the mucus layer, and immunological responses. Many in vitro models lack the mechanical and biochemical cues present in the living human gut, leading to discrepancies in absorption kinetics and bioavailability outcomes compared with clinical studies. This is particularly problematic for the evaluation of lipid-soluble vitamins and complex phytochemicals, which require micellization, transport, and hepatic first-pass metabolism for bioefficacy.

However, reproducibility and scalability have substantial limitations. Most models are lab-scale and not designed for high-throughput analysis, which is essential for screening multiple food matrices, processing conditions, and functional formulations in industrial R&D settings. Additionally, human inter-individual variability, such as age, gender, health status, and genetic differences, is inherently difficult to replicate in current models, limiting the generalizability of in vitro results for population-wide dietary recommendations. Finally, although integration with cell culture systems (e.g., Caco-2 and HT29-MTX) and organ-on-chip technologies has improved their physiological relevance, these systems are still costly, time-intensive, and technically demanding. Moreover, the materials used (e.g., dialysis membranes or synthetic chips) may adsorb nutrients or drugs, thereby introducing additional bias. In summary, although in vitro digestion models offer powerful tools for simulating gastrointestinal processes, their limitations must be carefully acknowledged. Future research should focus on enhancing physiological relevance, incorporating microbiota-host interactions, and aligning model outputs more closely with human clinical data to ensure the reliable prediction of nutrient bioaccessibility and bioavailability. Standardization efforts, open-access protocols, and cross-laboratory validations are also essential steps for improving reproducibility and scalability. Bridging the gap between in vitro models and clinical outcomes requires enhancing both the physiological realism and predictive value of digestion simulations. Recent efforts emphasize incorporating dynamic pH changes, secretion kinetics, peristaltic motion, and individualized gastrointestinal transit parameters to better reflect human digestive variability (Mulet-Cabero et al., 2020). Coupling digestion models with intestinal epithelial systems such as Caco-2 or HT29-MTX co-cultures provides insights into absorption, transport, and epithelial barrier interactions that cannot be captured by digestion alone. Integration with gut microbiota fermentation platforms such as SHIME allows the study of colonic metabolism and the formation of clinically relevant metabolites. Emerging computational tools, including physiologically based pharmacokinetic (PBPK) modeling and machine-learning algorithms, now enable the translation of in vitro bioaccessibility data into predictions of human plasma exposure ((Kamiloglu & Capanoglu, 2018; Y. Li et al., 2024). Together, these advances strengthen the translational relevance of in vitro digestion models and offer practical pathways to align laboratory findings with clinical and real-world nutrition data.

## 7 Future perspectives

As in vitro digestion models continue to evolve, several promising directions are emerging to enhance their translational relevance and scientific impact. One of the most urgent needs is standardization of methodologies across laboratories. Although the INFOGEST protocol has become a globally recognized benchmark, variability still exists in enzyme sources, concentrations, pH adjustments, and sampling intervals. Ongoing international collaborations aim to address these inconsistencies and enable reproducible and comparable results across studies and industrial settings. Personalized nutrition and precision health are poised to benefit significantly from in vitro simulation tools. Future models may incorporate individual-specific parameters, such as age, sex, disease state, genetic polymorphisms, and unique gut microbiota profiles. For example, integrating fecal inocula into colonic fermentation systems can better replicate host-specific responses. Such personalized models would be invaluable for assessing the digestibility and metabolic impact of customized diets and nutraceuticals. In an industrial context, the future lies in the development of high-throughput digestion screening systems. These platforms enable rapid evaluation of multiple food matrices, functional ingredients, and encapsulation strategies for bioactive compounds. Automation, miniaturization, and integration with omics technologies are crucial for achieving cost-effective and scalable digestion assays tailored to R&D pipelines. Moreover, the convergence of in vitro digestion models with machine learning and computational biology will allow more accurate predictions of nutrient bioavailability and physiological responses. This synergy could support regulatory submissions and accelerate functional food development with stronger scientific support. Despite these advancements, the limited correlation between in vitro and in vivo outcomes remains a major challenge. Future research must focus on validating the in vitro findings using clinical data. Longitudinal human trials, combined with biomarker tracking, will help bridge this gap and solidify the role of digestion models in nutritional and pharmaceutical sciences. In summary, future progress in in vitro digestion research will depend on stronger standardization, technological advancement, and closer clinical relevance. Looking ahead, advancing in vitro digestion research will require improved technical specificity, stronger standardization, and closer alignment with in vivo physiology. Although current static and dynamic models have been widely adopted, they still lack harmonized guidelines for enzyme activity, bile composition, pH control, and matrix interactions, which limits cross-study comparability. Emerging platforms such as 3D-bioprinted intestinal tissues and gut-on-a-chip systems offer promising solutions by integrating shear stress, mucus dynamics, epithelial renewal, and microbiome interactions. However, challenges remain regarding cost, technical expertise, long-term stability, and validation against human data. Future efforts should focus on establishing unified performance benchmarks, combining digestion models with physiologically relevant absorption platforms, and integrating computational tools for predictive modeling. These advancements will be essential for improving reliability, reproducibility, and the translational value of next-generation in vitro digestion systems.

## 8 Conclusions

In vitro digestion models have changed the landscape of nutrient release, transformation, and absorption studies, thus opening up cost-effective, ethical, and reproducible alternatives to in vivo experiments. Their journey has been from basic static systems through complicated dynamic models and gut bioplatforms, and this development has allowed these instruments to acquire significant global requirements for understanding the bioaccessibility and bioavailability of nutrients from different food matrices. The fusion of cutting-edge instruments and absorption modules, such as Caco-2 cells, organ-on-chip, and multi-omics platforms, has further amplified their potential to depict human gastrointestinal physiology. Several challenges remain to be addressed in vitro. One of these is the lack of standardized protocols across laboratories. In addition, there is limited gut microbiome simulation and, most importantly, weak correlations with human in vivo outcomes. Furthermore, problems concerning scalability, reproducibility, and regulatory validation hinder the scope of product development and regulatory assessments. Initiatives such as INFOGEST, the use of AI for modeling, and personalized nutrition frameworks are expected to bring the next generation of in vitro systems. Human clinical studies are essential for the validation and industrial application of these models in food innovation. In the long run, the modification and validation of in vitro digestion models will have a double effect: first, it will be a novelty in the study of nutrient functionality, and second, it will simplify the production of health-promoting, evidence-based food and nutraceutical products that are specially designed for precision nutrition and have a positive impact on global health.

## Consent of participate

Not applicable.

## CRediT authorship contribution statement

**Farhang Hameed Awlqadr:** Writing – review & editing, Writing – original draft, Supervision, Resources, Conceptualization. **Mohammed N. Saeed:** Writing – review & editing, Visualization, Software. **Othman Abdulrahman Mohammed:** Writing – review & editing, Visualization, Data curation. **Syamand Ahmed Qadir:** Writing – review & editing, Resources, Investigation. **Aryan Mahmood Faraj:** Writing – review & editing, Resources, Investigation. **Seyed Mohammad Najibi Hosseini:** Writing – review & editing, Resources, Investigation. **Muhammad Tayyab Arshad:** Writing – review & editing, Visualization, Validation. **Muhammed Adem Abdullahi:** Writing – review & editing, Visualization, Validation, Methodology. **Tablo H. Salih:** Writing – review & editing, Project administration, Funding acquisition.

## Consent to publish

Not applicable.

## Ethical approval

Not applicable.

## Funding

No funds or grants were received for this study.

## Declaration of competing interest

The authors declare that they have no known competing financial interests or personal relationships that could have appeared to influence the work reported in this paper.

## Data Availability

Data will be made available on request.
