# Supplementary material for: In vitro digestion models in food chemistry: advancements, challenges, and applications in nutrient bioaccessibility and bioavailability
Source: Food Chem X. 2026 May 6;36:103951. doi: 10.1016/j.fochx.2026.103951 (PMC13188131; doi:10.1016/j.fochx.2026.103951)
Supplement: Supplementary material [file mmc1.docx]

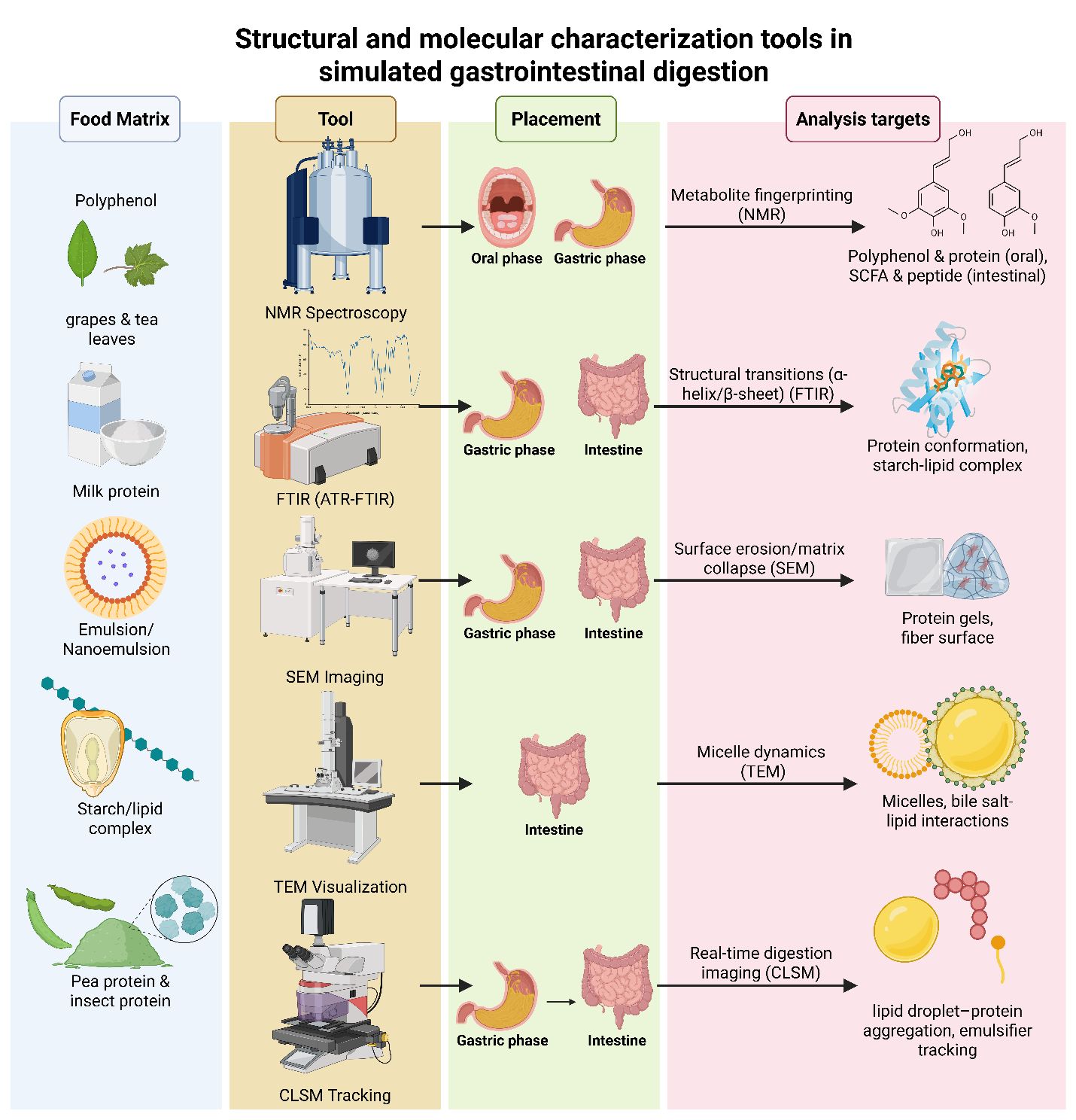


Figure S1 Structural and molecular characterization tools used in in vitro digestion studies across food matrices.


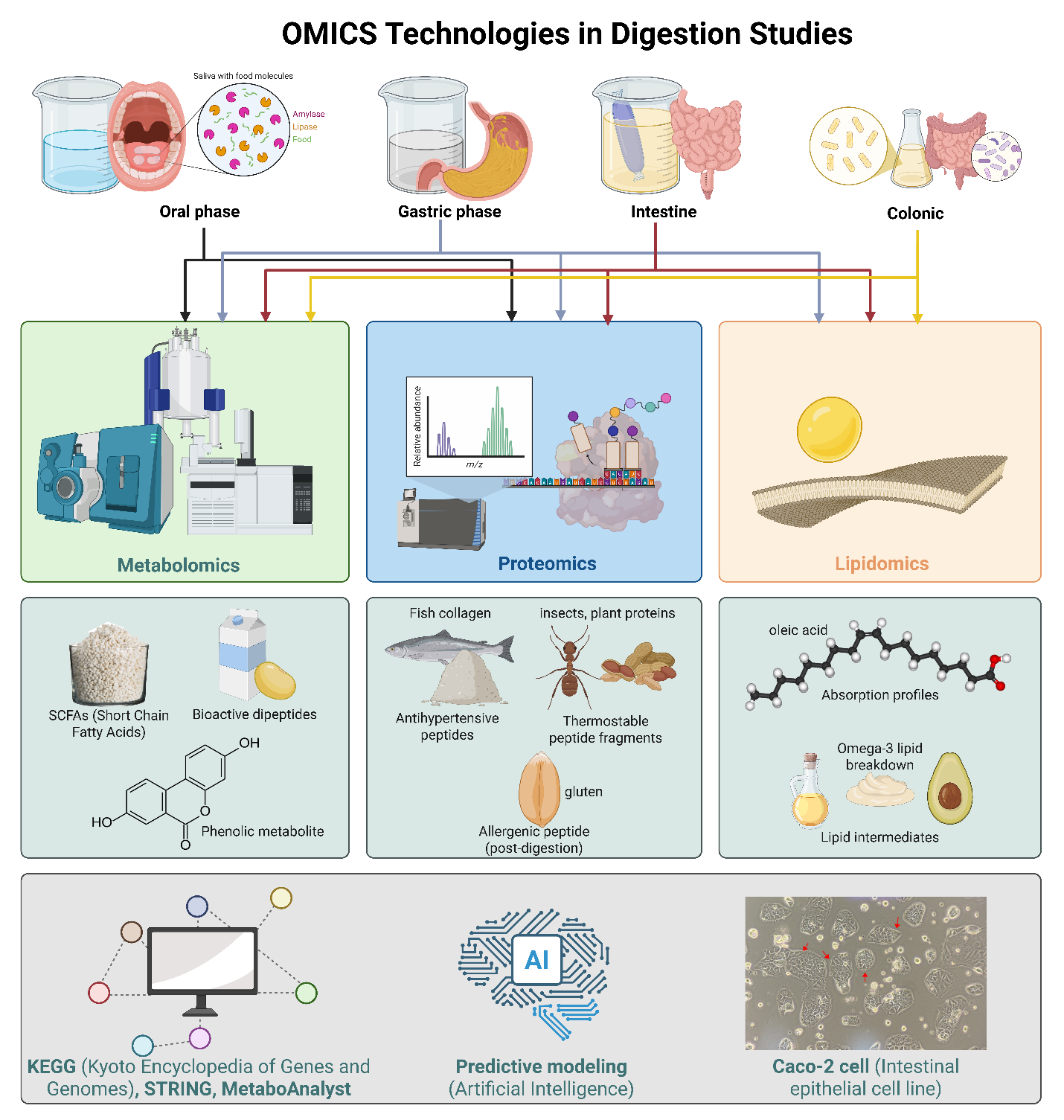


Figure S2 Combining OMICS technologies and in vitro digestion models to investigate nutrient metabolism and bioactivity.

Table S3 Applications in Nutrient Bioaccessibility and Bioavailability

| Nutrient/Compound | Delivery/Matrix Type | Enhancement Observed | Key References |
| --- | --- | --- | --- |
| Iron; Zinc | Cereal with ascorbic acid; wheat bran with phytase | Iron bioaccessibility increased through ascorbic-acid-mediated reduction, and zinc release increased by 30% after phytase treatment | (Shilpa & Jyothi Lakshmi, 2012) |
| Calcium Folate | Prebiotic fiber Fortified rice matrix | Calcium solubility increased through fiber-induced pH buffering, and folate stability was improved in the starch matrix during digestion | (Whisner et al., 2016) |
| Vitamin D; Anthocyanins | Lipid emulsions; berry matrices | Vitamin D bioaccessibility increased in emulsified form, while anthocyanin retention improved under lower pH conditions with associated antioxidant transformation | (Rabelo et al., 2018) |
| Vitamin B12; Docosahexaenoic Acid (DHA) | Zein nanoparticles Fish oil nanoemulsion | Vitamin B12 retention exceeded 85% during digestion, while DHA bioaccessibility and gastrointestinal stability were improved | (Wang et al., 2022) |
| Polyphenols; Resveratrol | Coffee matrix Casein micelles | Degradation was reduced, antioxidant transformation increased, and intestinal absorption improved in vitro | (Cheng et al., 2020; Tagliazucchi et al., 2010) |
| Curcumin; Beta-glucans | Alginate-chitosan beads; oat matrix | Curcumin showed five-fold higher intestinal bioaccessibility, while increased viscosity from beta-glucans slowed release and modulated glycemic response | (Silva et al., 2021) |
| Omega-3 fatty acids (EPA/DHA); Quercetin | Spray-dried microcapsules; lipid-based delivery | Retention exceeded 70% during digestion, and micellarization increased in the presence of dietary fat | (Jiang et al., 2024; Quintriqueo-Cid et al., 2024) |
| Lactobacillus rhamnosus; Soy Isoflavones | Hydrogel beadsSoy milk matrix | Survival increased by 40% under gastrointestinal conditions, and post-fermentation bioavailability improved | (Ferreira Leite Ladislau et al., 2021; Hati et al., 2017) |
| Milk peptides; Magnesium | Milk digestion; green vegetable blend | Digestion generated tripeptides with antihypertensive activity, and magnesium release increased under acidic gastrointestinal conditions | (Rutella et al., 2016; Vukic et al., 2017) |
| Starch; EGCG (Green Tea) | Cooked rice Nano emulsions | Glucose release decreased following starch retrogradation, while EGCG stability and absorption efficiency increased | (Fu et al., 2020; Saratale et al., 2018) |
| Proteins (Legumes); Protein Hydrolysates | High-pressure treated legumes Enzymatic hydrolysates | Digestibility increased by 30–50%, and peptide activity was retained or enhanced after gastrointestinal digestion | (Arnal et al., 2023; Gharibzahedi & Smith, 2021) |
| Carotenoids; Pea Protein | Surfactant-stabilized emulsions Fermented pea flour | Micellarization of lipophilic compounds increased, and digestibility improved after fermentation | (S. Li et al., 2024; Liu et al., 2024) |
| Vitamin C; Beta-carotene | Zein nanoparticles Encapsulated in starch | Stability increased during the gastric phase, and retention improved through microencapsulation | (Mahalakshmi et al., 2020) |
| Zein-encapsulated vitamins; Vitamin E | Zein nanoparticles Self-emulsifying drug delivery | Retention and delivery efficiency increased, while solubilization and gastrointestinal stability were improved | (Qi et al., 2024; Wang et al., 2022) |

**
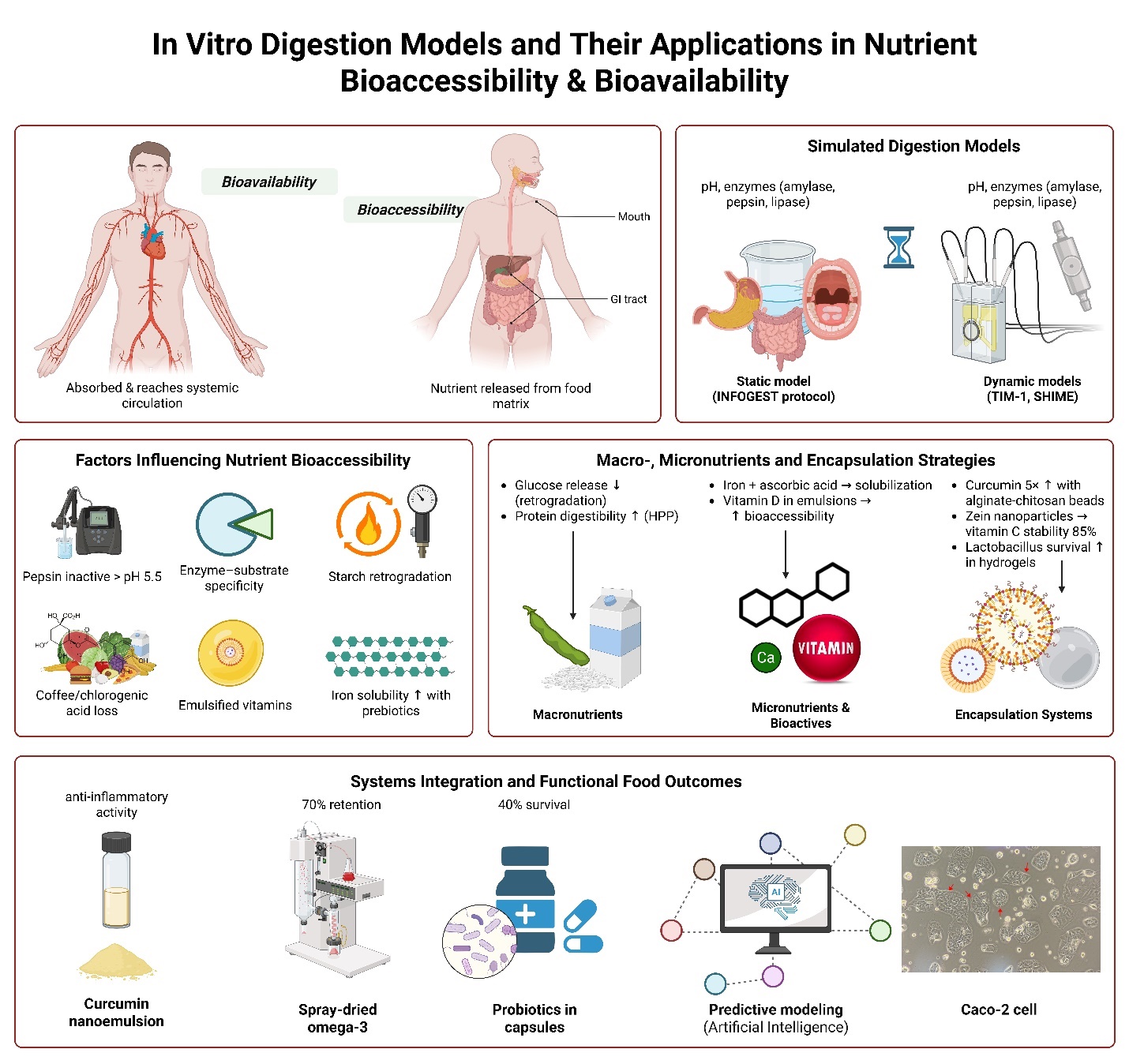
**

Figure S4 An overview of artificial digestion models and their functions in the evaluation of nutrient bioaccessibility and bioavailability.

**
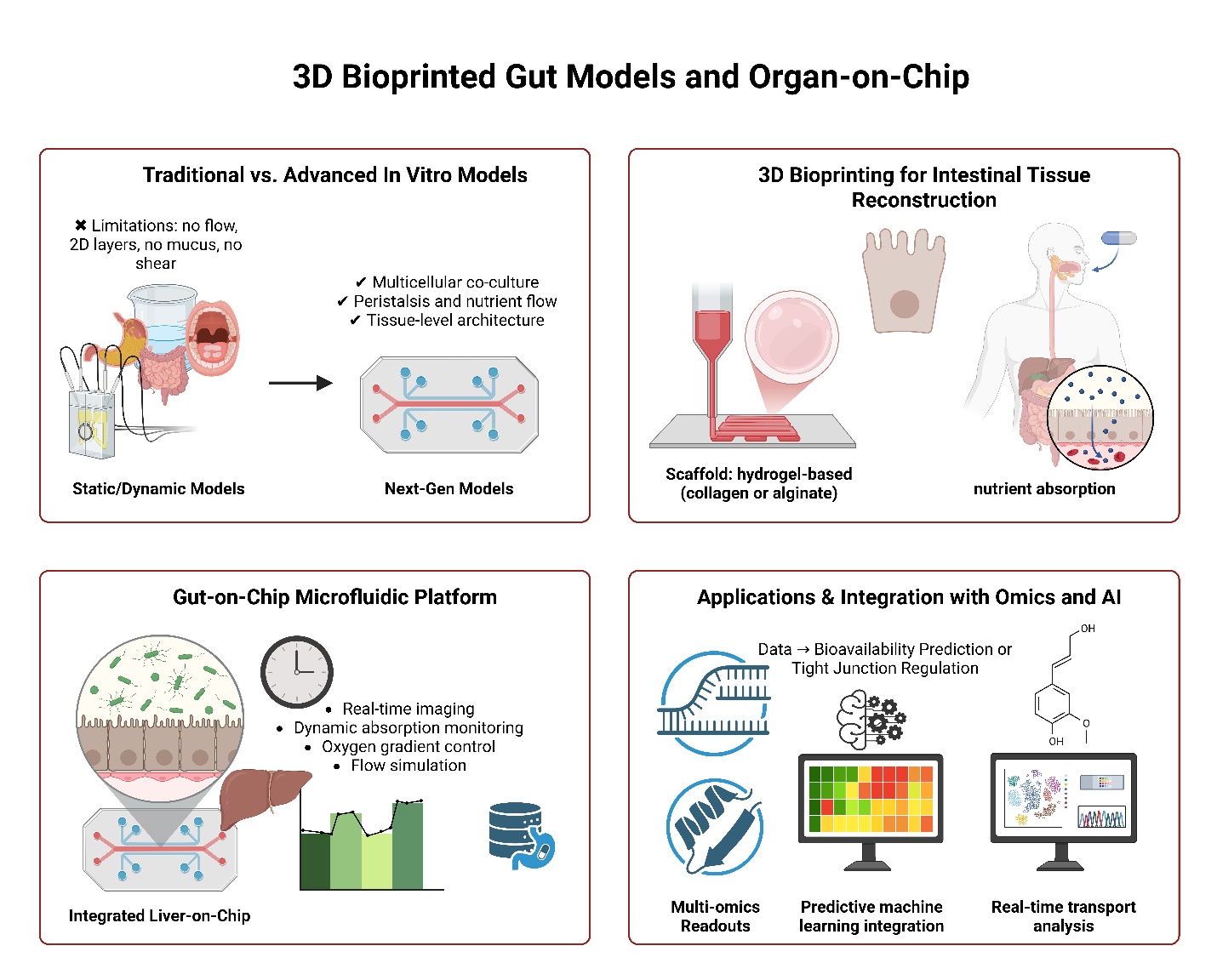
**

Figure S5 Next-generation in vitro platforms: 3D bioprinted gut models and gut-on-chip microfluidic systems.


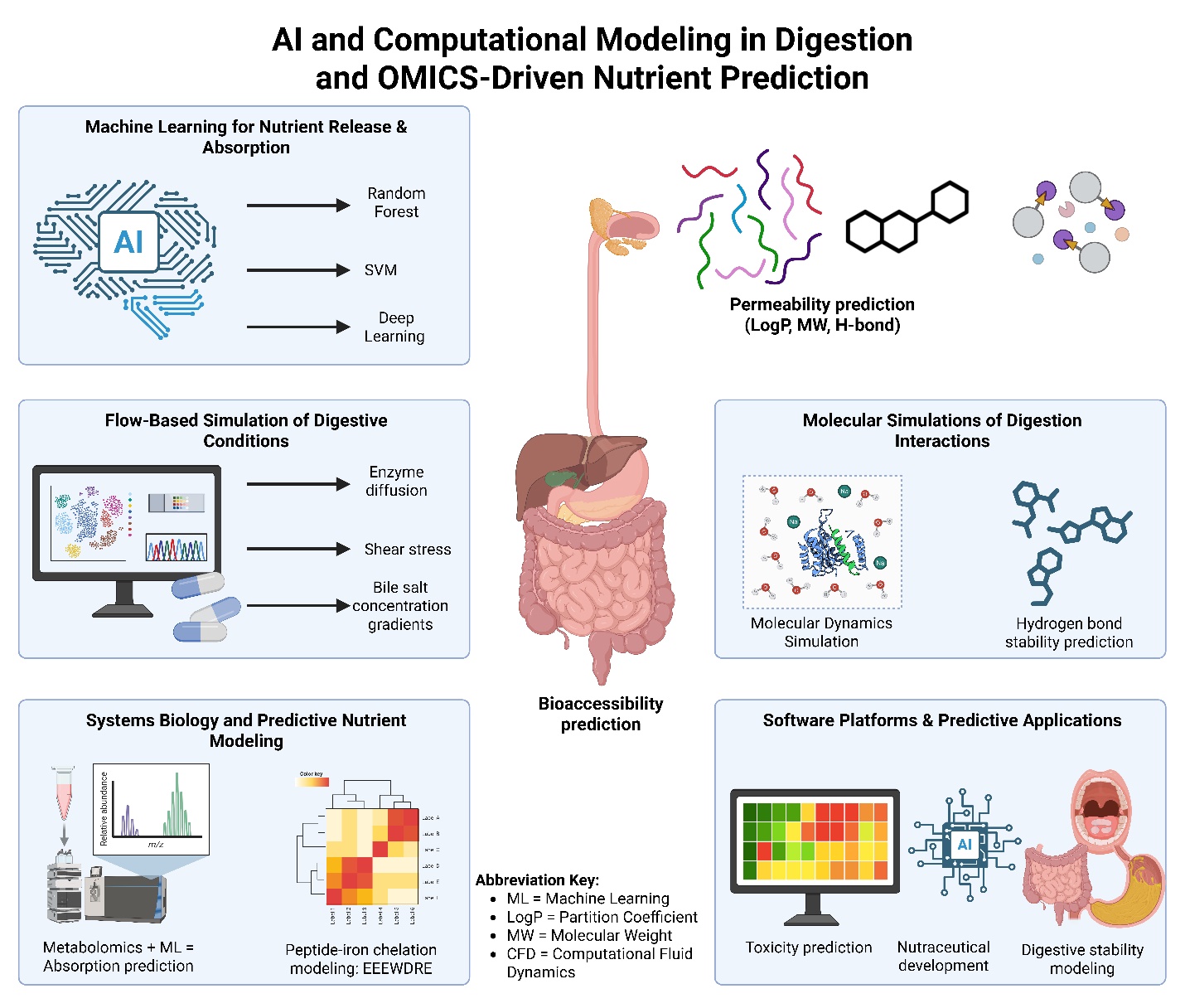


Figure S6 Integration of AI, computational modeling, and OMICS technologies in in vitro digestion and nutrient prediction.
